# Co-developing a framework to guide school-based substance use prevention (SSUP) interventions in Ghana

**DOI:** 10.1371/journal.pgph.0004345

**Published:** 2026-02-18

**Authors:** Abdul Cadri, Tracie Barnett, Tibor Schuster, Emmanuel Asampong, Alayne M. Adams

**Affiliations:** 1 Department of Family Medicine, Faculty of Medicine, McGill University, Montreal, Canada; 2 Department of Social and Behavioural Sciences, School of Public Health, University of Ghana, Accra, Ghana; Islamic Azad University South Tehran Branch, IRAN, ISLAMIC REPUBLIC OF

## Abstract

Substance use among young people is a global public health challenge with a high burden in African countries, including Ghana. Behavioural interventions implemented in schools can be effective in preventing substance use among young people in Ghana; however, these interventions are currently lacking. Evidence-based frameworks can contribute to the design and implementation of behavioural school-based substance use prevention interventions; however, to be useful, it is important that they reflect the culture and context in which the interventions will be implemented. The goal of the study was to co-develop a framework to guide the design and adaptation of school-based substance use prevention interventions in Ghana. A multi-method approach to develop the framework was implemented in five steps: 1) definition of scope and objectives of the proposed framework, 2) evidence review and synthesis of existing school-based substance use prevention interventions, 3) a mixed methods study of young people’s social networks in schools and its association with their substance use behaviour, 4) interest holder consultation (deliberative dialogue with 12 interest holders in Berekum, Ghana) to garner their recommendations for a school-based substance use prevention intervention in Ghana, and 5) framework iteration and its final visualization. The framework specifies what an ideal school-based substance use prevention should entail, components of the intervention, agents that can deliver the intervention, key interest holders to engage in intervention, and the application of theories, models, and frameworks in intervention design and adaptation. The SSUP framework provides a practical and flexible tool to guide the design and adaptation of school-based substance use prevention interventions in Ghana and similar contexts. It supports context-specific planning, interest holder engagement, and integration into existing school health promotion structure. Future research should explore its feasibility and effectiveness across diverse school settings. The framework offers a foundation for advancing locally grounded, prevention efforts across Africa.

## Introduction

Substance use among young people is a global public health challenge with significant burden, largely due to its increasing prevalence [1,2]. This burden contributes to the rising communicable and non-communicable diseases and injuries [3–5] in the World Health Organization’s (WHO) African region. Over the period 1990–2019, a 76% (CI = 70–79) rise in the prevalence of tobacco use was seen in Africa [6], with Ghana ranking among the countries experiencing the highest increase. Also, the World Drug Report [7] estimates that the prevalence of marijuana use in the West African region in which Ghana is located, is 9.4%, which is higher than the global average (4%). The World Drug Report also predicts that the prevalence of drug use in Africa will be close to 4 times (40%) the global average (11%) by 2030 [8]. The burden of substance use in Africa is worsened by the emergence of synthetic substances, including non-medical use of prescription opioids and cough mixtures; inhalation of glue, and petrol, among others [9].

As the substance use epidemic continues to evolve in most African countries, there is the need to adopt evidence-based approaches to prevention for vulnerable populations [9]. Ghana presents a compelling context for the development of substance use prevention framework due to the substance use epidemiological trends, policies, and school-based health infrastructure in the country. In Ghana, recent data has reported a high prevalence of substance use among young people [9]. A national survey estimated the past-month alcohol use among street-connected young people to be 74.7%, past-month cigarette and marijuana use were 68.4% each, whereas the reported lifetime illicit drug use was 24.5% [10]. Moreover, a survey among school-going adolescents in the Central region of Ghana estimated the prevalence of alcohol use to be 42% [11]. Recent studies also indicate the use of synthetic substances and non-medical use of prescription drugs among young people [12–14].

Ghana’s progressive drug policy landscape- the Narcotics Control Commission Act, 2020- emphasizes a public health approach to substance use. Specifically, it makes provision for prioritizing prevention, treatment, and harm reduction over criminalization [15]. This policy shift aligns with global calls for evidence-based prevention and positions Ghana as a leader in West Africa for reforming national drug policy framework.

Furthermore, Ghana has a comprehensive School Health Education Programme (SHEP) which serves as a platform for life skills training and school-based health promotion for young people [16]. However, structured and evidence-based content on substance use prevention remains limited in the SHEP. Given the existing SHEP structure and growing cross-sectoral engagement, Ghana offers a unique opportunity for developing and scaling school-based prevention strategies grounded in behavioral science and contextual realities.

Behavioural interventions, including substance use prevention interventions, offer evidence-based strategies with the potential to lessen the substance use burden among young people [17]. Substance use prevention interventions primarily prevent the onset and/or reduce the prevalence of substance use [18]. They can be implemented in multiple settings such as family, school, and community [19]. Schools have been reported to be an appropriate setting for the delivery of these interventions for three main reasons: (i) four out of five people who use substances begin before adulthood, (ii) schools offer a dynamic and efficient way of reaching many young people, and (iii) schools can adopt and enforce a broad spectrum of educational policies [20].

While several school-based substance use prevention interventions exist, they have mainly been developed and implemented in high income countries [21,22]. Among interventions implemented in low- and middle-income countries, only a few (8%) have been implemented in the African region [23]. Interestingly, those implemented in African countries- specifically Nigeria [24] and South Africa [25] – have demonstrated effectiveness in preventing the initiation of substance use and reducing the prevalence of substance use among young people. While these efforts are relevant to the Ghanaian settings, existing school health programs lack contents on substance use, indicating gaps in comprehensive school-based substance use prevention interventions in Ghana [23].

The design and adaptation of school-based substance use prevention interventions includes the development or modification of components deemed essential for intervention functioning [26], identifying and adjusting their nature or intensity, and the application of appropriate guidance around intervention implementation [27]. To enhance efforts towards school-based substance use prevention interventions, researchers recommend the use of evidence-based frameworks that are culturally appropriate to guide intervention design and adaptation [28].

Despite their relevance, there are no culturally appropriate, context specific evidence-based frameworks to guide the design and adaptation of school-based substance use prevention interventions in Ghana. In this study, with the input of Ghanaian youth and other interest holders, we co-develop a culturally appropriate, context specific framework to guide the design and adaptation of school-based substance use prevention (SSUP) interventions in Ghana.

## Methods

### Study design

We adopted a multi-method approach towards the development of the SSUP framework. Guided by the UNODC [29] recommendations, we meaningfully engaged young people in the framework design, and ensured that their participation in the project was accessible, safe, and relevant. The priorities outlined in this framework have been informed and supported by a range of interconnected strategies, including peer-reviewed evidence, grey literature, a school-based survey of young people’s social networks and substance use behaviours, as well as input gathered through a deliberative dialogue with interest holders. Also, we adopted the use of the term “interest holder” in this paper due to recent arguments that the use of the term “stakeholder” reinforces negative connotations in research, notably referencing colonialist practices, and can represent people who are affected by the outcome but have no power to influence it. The five sections below outline the steps we took in producing the SSUP framework:

#### Step 1: Define the scope and objectives of the SSUP framework.

This framework was intended to guide the design and adaptation of school-based substance use prevention interventions in Ghana, with potential applicability to other African countries with similar contexts. Its intended users include researchers, program planners, implementers, and policy makers in the substance use prevention field. A key tenet of the development process was to promote engagement of young people and other key interest holders, such that the framework produced would be informed by the lived experience of young people; culturally appropriate and relevant; adaptable to specific contexts; and implementable through collective efforts of multiple interest holders. During the conceptualization phase, we conducted a preparatory review of literature and found social networks to be a predominant risk factor of young people’s substance use behaviour [30], and also found that substance use among young people have important gender differences [31–33]. As such, we explored strategies to understanding and addressing them in the framework.

In line with theories, such as the normalization process theory [34], that explains the “work” needed to implement complex interventions, we needed to: (i) situate this framework as distinct from standard substance use prevention practices by establishing a comprehensive approach that includes prevention of substance use and treatment for substance use disorders; (ii) consider various strategies, including those that enable a gender-responsive, social network approach to substance use prevention; (iii) identify critical interest holder needs; and (iv) describe the necessary steps and processes to be considered when applying the SSUP framework for substance use prevention interventions for young people.

#### Step 2: Evidence review and synthesis.

As a next step, we conducted a scoping review of literature [23] to map the key components of school-based substance use prevention interventions in low-and-middle income countries (LMICs). The population of interest were young people in the age range 10 and 24 years. The outcomes of interest were *prevention of initiation of substance* or *reduction in prevalence of substance use* or both. We focused on randomized and non-randomized trials conducted in Low- and Middle-Income countries (LMICs) to maximize the relevance of the findings to Ghana, and potentially other African settings. The review also mapped the theories, models, and frameworks (TMFs) used in school-based substance use prevention interventions. We conducted the search using 8 bibliographic databases. Retained articles were analyzed using a descriptive content analysis. Based on this analysis, we identified six key components of school-based substance use prevention interventions in LMICs: education, peer leader, school environment, school policy, counselor, and guardian components.

#### Step 3: School-based survey of young people.

Since social networks, as well as gender, were important dimensions of the SSUP framework based on the brief literature search in step 1, we conducted a mixed methods social networks study (explanatory sequential mixed methods design) in two randomly selected schools. Both schools are situated in Berekum municipal, a mid-size city in the central Bono Region of Ghana, selected due to reports that substance use prevalence was high in the municipality [35]. We used a bounded social network approach; hence, students were asked to select their friends within the classroom. Also, all students in three selected classrooms were eligible to participate in the surveys. A total of 129 young people (79 males and 50 females) completed the quantitative self-administered paper-based survey, and a sub sample -25 young people (13 males and 12 females)- were selected for the qualitative in-depth interviews to elucidate the quantitative findings. The age range of the participants were 16–22 years. The survey explored the friendship networks of young people in school, including approaches to forming friendships, mechanisms of substance use influence in friendship networks, and the effect of existing gender roles and relations on substance use behaviour. The social network data was collected using an adapted Swiss student life codebook, a previously validated instrument [36]. We analyzed the quantitative data using descriptive (mean, frequency, percentages, and network graph visualization) and inferential statistics (exponential random graph modelling). The data was analyzed using R studio of R statistical software and Visone for the graph visualization. We also analyzed the qualitative data using the framework method, a structured form of thematic analysis [37]. Two members coded the qualitative data and all team members reviewed them to assess relevance and coherence. Key findings include gender identity being as a strong predictor of friendship network formation and substance use norms of a friendship network, as well as traditional gender norms has an influence on individuals’ substance use behaviour. Through the mixed methods study, we gained an understanding of how young people’s friendship networks may be associated with their substance use behaviour, as well as specific gender norms that may influence their substance use behaviour. The findings offered insights on key dimensions [network norms and gender norms) that could be leveraged in school-based substance use prevention interventions. The findings of this survey is being published elsewhere (Topic: *The peer dimension of young people’s substance use behaviour: a mixed methods social network analysis among senior high school students*).

#### Step 4: Interest holder consultation and feedback.

In the fourth step, we conducted a deliberative dialogue workshop with young people and adults to garner their recommendations for a school-based substance use prevention intervention, including components, delivery agents, and interest holders that are relevant to their local context. In deliberative dialogues, evidence is critically examined, and ideas, values, and priorities are exchanged in a manner that contributes to shared cognitive space or mutual understanding of an issue, which may facilitate the translation of evidence into action [38]. We recruited participants (July 9, 2023 to September 30 2023) using a purposive maximum variation sampling approach to ensure a diversity of backgrounds and experiences, and that different needs and perspectives were considered [39]. Recruitment was facilitated by our local community partners.

One week prior to the deliberation, all participants were given copies of a lay summary of the research findings from the literature review (steps 2) and mixed methods social network survey (step 3). Given the power imbalance between young people and adults in Ghanaian culture, three strategies were adopted to create a safe and inclusive space for equitable youth participation. First, prior to the dialogue, a capacity building workshop was organised for the young people to prepare them for a constructive dialogue with adult participants, and to empower them as valued voices at the table. Adult participants were also informed on how they could make youth participants feel safe and give them the opportunity to share their opinions. After the dialogue, we met the young people separately to give them the opportunity to reflect and provide feedback on the challenges they experienced in making their views heard during the deliberative dialogue process. They were also given the opportunity to make further recommendations in the separate dialogue with them only. The facilitators also used a roundtable discussion technique to ensure that young people at the table shared their opinion.

The dialogue was held on October 9, 2023 [10am to 12pm GMT) in a conference room at the Berekum Municipal Education Directorate. The session started with a brief presentation of the findings from steps 2 and 3, followed by a question-and-answer session. The dialogue was facilitated by the first author (AC), as he has the requisite training in deliberative dialogue facilitation. At the beginning of the session, common house rules were discussed [38], including respect for other people’s opinions and our collective commitment to confidentiality.

The dialogue had two phases: one separate small group discussion involving 6 participants each (consisting of individual reflection, group discussion, and group prioritization) and one whole group discussion with all participants. The guiding question for the dialogue is presented as a supplementary file (S1 File). Each group had a trained rapporteur (research assistant) who reported a summary of their group’s decisions and priorities to the whole group for further deliberation, while allowing other members of the group to add to their group’s summary. The discussion was held in a Ghanaian local language (Twi) and was audio-recorded.

The recorded deliberations were translated and transcribed verbatim from the local language to English by research assistants. The recorded interviews and transcripts were then validated for accuracy by the first author, since he is proficient in both languages. We analyzed the data inductively using a Framework Method [37], a structured form of thematic analysis that involves the creation of data displays to facilitate the identification of themes and patterns in the data. Following the steps of thematic framework analysis, we first familiarized with data by listening to the recorded interviews and reading the transcripts. Second, inductive codes (created using NVivo 12) linked to the research questions were created. Third, a thematic framework was identified, and related codes were indexed. In the fourth stage, we created a matrix, and the data were charted into the matrix with supportive quotes. We then interpreted the data using an analytic memo and discussion among team members. Themes were identified by AC and reviewed by AA to assess relevance and coherence, and the final themes and illustrative quotes were reviewed by all the research team.

#### Step 5: Produce a specification of the framework.

Following the deliberative dialogue, the framework was produced using the findings and additional literature review. This convergence of literature review, survey data and research and interest holder consultation informed the final specification of the SSUP framework.

### Ethical considerations

The ethical approach of this study took into consideration informed consent, voluntary participation, and respect for participants, including confidentiality and protecting respondents’ privacy. Ethics approval to conduct this study was granted by the Kintampo Health Research Centre (KHRC) Institutional Ethics Committee, Ghana (*KHRCIEC/2023-09*) and the Research Ethics Board of the McGill faculty of Medicine and Health Sciences (*A01-B104-22A/ 22-07-110*). We also sought approval from the Director of Education in the municipality in which the study was conducted, as well as the head teachers at the participating schools.

Written informed consent was sought from participants, and they were made aware that their participation should be completely voluntary, and they can opt out of the study at any time. Also, the informed consent specified the study purpose and procedure, potential risks and benefits, and compensation for participants. For emancipated minors, parental consent was sought, followed by assent. All participants signed the consent form before participating in the dialogue.

## Results

### Deliberative dialogue participants

A total of twelve participants engaged in the deliberative dialogue. This included 4 young people (2 boys and 2 girls) and 8 adult participants. The adults included 3 trained teachers, 2 community mental health officers, 1 Non-Governmental Organization (NGO) partner, the Municipal SHEP coordinator, and the Municipal Director of Social Welfare and Community Development. In terms of gender, 5 participants were female whereas 7 were male. Equal number of young people from each school participated in the dialogue (1 boy and 1 girl from each school). All participants were from the Berekum municipality.

### Priority areas and recommendations for school-based substance use prevention interventions

**Fig 1:**
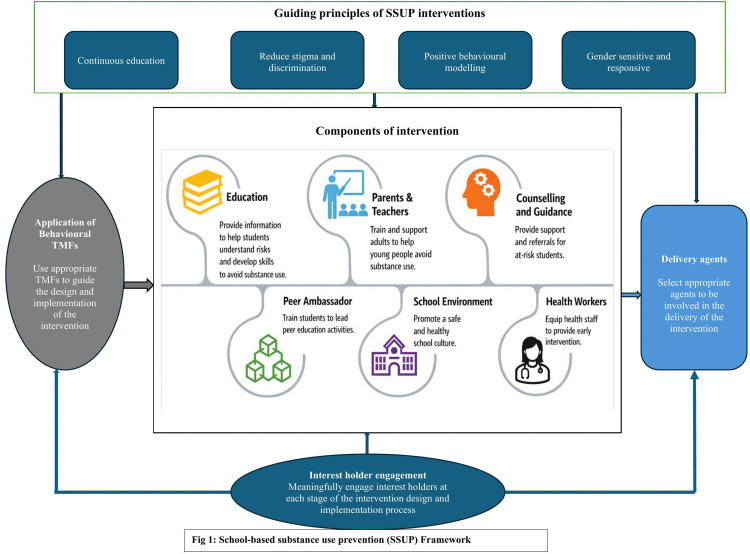
School-based substance use prevention (SSUP) Framework.

The process of deriving this framework involved presenting the participants with evidence from the literature review in step 2 and the mixed methods social network analysis in step 3 of the methods, and asking them to propose add-ons, suggest alternatives, and share their perspectives on the feasibility of various strategies in their setting. Based on the evidence presented and their own experiences, key interest holders suggested four major priorities in small and large group deliberations. These priorities included what an ideal SSUP intervention should entail, components of the intervention, agents that can deliver the intervention, and other key interest holders to engage in designing and implementing the intervention (Fig 1: School-based substance use prevention (SSUP) Framework). Further consideration of results from the scoping review, the social network survey, and additional literature search resulted in the application of behavioural theories, models, and frameworks (TMFs) as another priority dimension of the SSUP framework.

### An ideal school-based substance use prevention intervention

Based on interest holders’ recommendations, as well as synthesis of the findings from the mixed methods study in step 3 and further grey literature search, we derived four core guiding principles of a school-based substance use prevention intervention: continuous education, reduce stigma and discrimination and be supportive, positive behavioural modeling, and gender sensitivity.

#### Continuous education.

There was broad recognition among the interest holders that an ideal school-based substance use prevention intervention in their setting would be meaningful if it includes continuous education on substance use. Some key interest holders recounted events where education on substance use were organized once in a semester or year in schools, and that may not be enough to effect behaviour change.

*“I suggest the intervention should include regular education on substance use, not just once but consistently…”* (Participant C, teacher).

Verplanken and Orbell [40] offers an underlying explanation on the relevance of continuously performing a desired action (*continuous substance use education in this context*). Based on their assertion, continuous education on substance use may result in a newly formed behaviour of not using substances which can lead to formation of habit on substance use prevention. This habit is a memory-based propensity derived from cue-response associations in memory and acquired through repeatedly acting in response to those cues in a stable context.

Indeed, through continuous education, interventions could maximize long-term substance use preventive behaviours by making desirable substance use prevention behaviours habitual, so that they acquire features such as persistence or insensitivity to counter-information [41]. In practice, continuous education requires interventions to consistently provide education on substance use prevention throughout the duration of the intervention. For example, a 13-week school-based substance use prevention intervention provided 13 consecutive 45-min lessons to students with one lesson each week [42]. Another school-based substance use prevention intervention’s [43] approach to regular education involved organizing a classroom education session once or twice a month throughout the duration of the 1-year intervention.

While there isn’t a definitive approach to deciding the number of educational sessions, frequency (daily, weekly, bi-weekly, monthly, etc.) of sessions, and duration (30 minutes, 60 minutes, etc.) of each session, they are typically determined by the duration of the entire intervention (1 school term, 1 school calendar year, 2 school calendar years, etc.), the number of topics to be covered, and the appropriate fit within the school’s existing workflows, system, and processes. Most importantly, the number of sessions, frequency, and duration of each session can be modified based on the needs of the target group and ongoing feedback.

#### Reduce stigma and discrimination and provide support.

People who use substances face discrimination and stigma (a set of negative attitudes and stereotypes) that can impact their health and wellbeing physically, psychologically, and socially [44]. Particularly in a setting like Ghana where substance use is perceived as a morally unacceptable behaviour, people who use substances may experience stigma, discrimination and a lack of support [45]. Findings from the mixed methods social network study (step 2) showed a high prevalence of substance use among school-going young people. As such, interest holders recommended that school-based substance use prevention interventions should aim to reduce substance use related stigma and discrimination to facilitate participation of young people who use substances. They also indicated that young people who have initiated substance use may need comprehensive social and medical support to be able to quit; hence the suggestion that provisions be made as part of the intervention to provide the needed support, including counselling services. This approach conforms with recommendations and guidelines issued by the UNODC [46].

*“From the experience that I have, all those who into drug use, they understand that they are doing the wrong thing so, we must show love and compassion to support them. We should avoid discrimination and stigma.”* (Participant B, NGO).

Substance use related stigma and discrimination can occur at the individual level (e.g., internalized stigma), interpersonal level (e.g., use of derogatory terms or dehumanizing labels), and structural level (e.g., unequal access to opportunities) [47]. It is important that substance use stigma and discrimination reduction strategies are incorporated into the SSUP intervention’s components and activities, including language and words used in intervention delivery. Stigma and discrimination reduction strategies such as skill-based training, education, and resource provision (e.g., language guides) [47] are therefore recommended to be included in intervention components. This includes the replacement of stigmatizing language with preferred, empowering language that doesn’t reinforce negative connotations [48]. The National Institute on Drug Abuse [49] and the Recovery Research Institute [50] offer a range of useful, non-stigmatizing words that can be used in the design of SSUP interventions.

#### Positive behavioural modeling.

The influence of friendship networks on individual substance use behaviour is well established in the literature [30]. Findings from the social network study (step 3 of project methods) indicate that young people’s friendship network norms offer opportunities for behaviour imitation, thus, functioning as an underlying mechanism of influence on substance use behaviour. In recognition of this, interest holders recommended that an ideal school-based substance use prevention intervention should adopt an approach that will reduce negative influence in young people’s friendship networks. They recommended the encouragement of positive behavioural modeling as an approach to overcome network norms that influences substance use.

*“One way we can deal with the negative influence of friends substance use is to encourage positive role-modeling among friends*”. (Participant F, young person).

This recommendation aligns with the social learning theory that suggests that behaviour of young people can be shaped by their peers through modeling and social reinforcement [51]. Using this approach, researchers, program planners, and implementers are encouraged to incorporate strategies of positive behaviour modeling into the components of the school-based substance use prevention intervention. Thus, the components can include strategies that provide young people opportunities to create healthy friendship networks; provide opportunities that foster the adoption of positive coping skills from their peers; and teach them refusal skills to resist peer pressure and negative network influence [52,53]. In behaviour modeling training, it is recommended that young people are given the opportunity to practice the newly learnt skills, followed by feedback and reinforcement of skills.

#### Gender sensitivity and responsiveness.

Results from the social network survey (step 3) revealed a higher prevalence of substance use among boys. Interest holders also noted popular sociocultural masculinity traits which normalize substance use among boys. Historically, substance use prevalence has been higher among boys compared to girls [54–56]; however, more recently, an increasing trend is being reported among girls [57]. Calling attention to this trend, interest holders indicated the need to adopt approaches to address the growing prevalence and burden among both girls and boys. They recommended that school-based substance use prevention interventions be designed to include a gender dimension that addresses the unique needs of boys and girls.

*“... I think in the explanation, they say only men should use drugs, but it shouldn’t be that way. I would like them to put it in a better way that all the gender shouldn’t use drugs at all. We have to find ways to educate both boys and girls in a way that is appropriate for them all.* (Participant I, community mental health officer).

These recommendations are supported by recent calls for gender sensitive and gender responsive approaches in substance use prevention interventions [57]; specifically, a call for intervention to consider the influence of gender roles, norms, and inequalities; incorporate strategies to raise awareness about gender inequalities; and adopt measures to address them in intervention design. Drawing on WHO guidance on designing gender sensitive and responsive programmes, strategies relevant for the design and implementation of school-based substance use prevention interventions [58] include: (i) integrating gender analysis and gender responsive actions into intervention design; (ii) building the capacity of programme staff and stakeholders, including peer leaders, to address gender inequalities; (iii) promote meaningful participation of women in various phases of the intervention; and (iv) address gender in the monitoring and evaluation of SSUP interventions.

The need for gender sensitive and responsive approaches in SSUP interventions is further necessitated by the gendered nature of youth friendship networks, apparent in youth preferences for friendship and activities with peers of similar gender identity. Findings from our social network study indicated that similar gender identity is a strong predictor of selection of friends, thus, females generally prefer their fellow females as friends, and males mostly prefer their fellow males as friends. Based on young people’s preferences, when designing SSUP intervention activities involving group interactions, the consideration of gender is key. For example, activities that involve sensitive discussions can be organized in separate groups of males and females. Interest holders further indicated the need for interventions to shift the mindsets of young people from traditional gender roles and traits by engaging in activities that encourage then to question traditional gender assumptions and expectations, and their adverse influence on substance use behaviour.

### Components of a school-based substance use prevention intervention

The interest holders recommended a multi-component approach to the SSUP intervention. Based on their insights, experience, and deliberations of findings from the scoping review in step 2 and social network study in step 3, we identified 6 components that can inform the design of effective SSUP interventions: education, parents and teachers, counselling, peer ambassadors, school environment and health workers.

#### Education.

The interest holders identified education as a key component of school-based substance use prevention intervention. The use of education in substance use prevention interventions is also recommended by international organizations, including the WHO. The WHO Framework Convention on Tobacco Control outlines education on harms related to tobacco use as a prevention strategy [59] which is relevant to use of other substances. Indeed, many substance use prevention interventions for young people employ education as a behaviour change technique [23].

*“Education should be key in the schools... we should educate them on dangers of substance use, peer adolescent influence, public education, and encourage student research. That is all”*. (Participant A, social welfare and community development)

According to Bukoski [60], several approaches to substance use education exist, one of which is an information approach whereby scientific evidence is provided on how the use of substances affects the brain [60]. This approach is defined as a persuasive education strategy and is mostly used with the goal of increasing the target group’s knowledge and changing their attitudes towards substance use. A range of topics can be considered when applying this approach in SSUP interventions. Common topics include short- and long-term effects of substance use, myths and facts about substance use, monetary costs of substance use, recognizing peer and social influence, substance use policies and laws, impacts on relationships and family, among others [23,61]. Given the impact of gender on substance use, it is important to include relevant topics on gender and substance use in the educational component. Specifically on tobacco use, the WHO has recently encouraged education on the tobacco industry’s exploitation of young people which includes raising young people’s awareness of strategies such as e-cigarette flavours and tobacco concerts, as manipulative tactics to get young people hooked on their addictive products [62]. Notably, most young people who did not use substances during our in-depth interviews cited knowledge on harmful effects of substance use as protective factor.

Another educational approach indicated by Bukoski [60] is affective education, often regarded as a skills-based approach. In this approach, underlying personal characteristics such as self-esteem, decision making, communication, are targeted. Indeed, capacity building approaches can be used to enhance skills which include problem solving, resistance to peer pressure, coping strategies, anger management, interpersonal relationship, assertiveness, refusal and quitting, goal settings, and self-management [23,61]. Researchers, program planners, and implementers of SSUP interventions can combine information and affective approaches by complementing the provision of information on relevant topics with relevant affective approaches [23].

It is important to choose topics that best address the risk factors of substance use for a given context and target population. Topics should be appropriate to the implementation site, type of substance(s), the age and gender group of interest. Lastly, it is recommended that the educational component creates an enabling environment and maximize opportunities that enable young people to use mobile health technologies [63], that are self-directed, non-stigmatizing, and effective in providing needed information and skills, as well as links them to appropriate support.

#### Parents and teachers.

Interest holders pointed to the importance of parents and teachers as a component of SSUP interventions. Effective parenting has been identified as one of the protective factors of substance use among young people [64,65]. Indeed, findings from interviews with young people in step 3 of the study revealed that most young people who did not use substances cited parental advice and control as motivating factors to not starting or to quitting substance use. However, some interest holders believe that parental engagement alone may be insufficient, given that young people spend substantial time in school (especially boarding students). In these circumstances, teachers play a vital role in educating and supporting young peopleon substance use prevention.

*“Both parents and teachers should be involved in the program. For the parents, they should be involved because even the bible says that we are to train up a child the way he should grow so that he will never depart from it... But sometimes the parental control is not effective and so, teachers’ involvement would be better”*. (Participant E, Policy maker).

Parents and teachers are regarded as socialization agents of change who are well positioned to provide parenting and teaching techniques that reinforce attitudes and behaviours to address young people’s vulnerabilities [66]. Within SSUP interventions, this component enhances the supportive and educational roles of parents and teachers through the provision of skills and knowledge.

A key approach in this component is to train teachers and parents on communication and positive role modelling, as well as raise their awareness on relevant substance use topics such as trends, risk and protective factors [67]. In response to interest holders’ perceptions that parents and teachers may discriminate against young people who use substances, the training can emphasize their role in supporting young people who use substances and communicating with them in an open and non-judgemental manner [68]. Whether through workshops, online webinars, or factsheets, interest holders also emphasized that training be informed by evidence-based practices to assist parents and teachers in managing situations when substance use is suspected. This includes looking out for behaviours that indicate the use of substances among young people. Petras [69], for example, suggests training parents and teachers on how to observe and interpret young people’s cues within specific contexts, such as the way they manage anger control or respond to certain tasks assigned to them. They also recommended regular check-ins with young people regarding their wellbeing and social environment, instead of waiting for issues to arise before having a conversation with them.

Lastly, building community networks for parents and teachers as part of this component may be an important source of support [70], providing opportunities for parents and teachers to network, share challenges and navigate solutions together. A peer network of parents and teachers will help promote a sense of collective effort and minimize feelings of isolation when dealing with young people’s substance use behaviour.

#### Counselling.

Another component of SSUP interventions identified by interest holders was the provision of school-based counselling, especially for young people who are already using substances harmfully, or those addicted to substances. For the latter group, the counselling component will assist them in quitting and dealing with withdrawal, and in developing skills to aid in recovery. This component is in line with the recognition of substance use addiction as a complex medical condition that requires multiple intervention strategies, including talk therapy or psychotherapy [71].

*“About counselling. It’s very important component. Because the counselors can be closer to the students and get more details about the students more than we the teachers. Some students find it very difficult to share their secrets with their teachers, but they can share it with the counselor because they know how to do that well”*. (Participant K, teacher).

In many settings, including our study setting, harmful substance use and addiction may be seen as moral issues; hence, victims may experience stigma and discrimination which discourage help seeking [45]. Based on these circumstances, interest holders noted the role of counselling and guidance teams in providing access to needed support, given that young people might find it easier to open up to counsellors compared to peers, teachers or guardians. They also recognized that counsellors are better equipped to help young people identify risk factors of substance use and relapse, as well as enhance their resistance skills.

Several strategies to offering school-based counselling exist. Individual counselling is especially useful to students who are currently using substances or at high risk of substance use, whereby confidential and personalized support is provided based on the young person’s circumstances and includes them in developing an intervention plan and setting goals towards recovery [72]. Group counselling is another strategy in which the counsellor provides guidance to small groups of students with similar experiences and creates opportunities for them to learn from and support each other [73]. In settings where professional counselling teams aren’t available in schools, linkages with counsellors or mental health service providers in hospitals and health facilities will be needed to ensure access to the needed support.

#### Peer ambassadors.

Interest holders recommended the establishment of substance use prevention clubs in schools and task some students to be ambassadors of substance use prevention. Under this component, “peer leaders” are identified and trained on substance use prevention messaging, thus equipping them with skills needed to support fellow students in eschewing harmful substance use. Interest holders’ reasons for recommending this approach was that young people know themselves better; hence, they will best know whom to target.

*“…What we need to do is to create an educational program to get more students to be trained to understand what we are doing. So that they will do person to person contact. The students will know best which of their colleagues is into substance use. so, if we get student ambassadors in schools and train them, they will go to their colleagues and speak to them to address substance use problems”.* (Participant D, young person)

Peer leaders have been predominantly used insocial networks interventions [74,75]. The Centre for Disease Control guidelines on implementing school health substance use prevention programs recognize peer leaders to be important components of prevention programs [76]. Harnessing the power of social influence [60], this approach recognizes peer norms as an important risk factor for substance use and focuses on correcting perceived social network norms and enhancing peer resistance skills [77].

Notably, in this study, a “friendship network norm” was found to be the underlying mechanism of network influence on young people’s substance use behaviour, i.e., young people tended to use more substances in friendship networks that accept substance use and regard it as normal and vice versa. As such, both prosocial norms and norms that oppose the use of substances in friendship networks are encouraged in the peer ambassador component. Most interventions that use this approach have been deemed “model” programs and shown promising results [78]. They adopt a common strategy by selecting and training peer leaders who are supported to teach substance use resistance skills to their peers that includes encouraging them to quit substance use or not start using substances [23].

A second social network approach is the segmentation approach in which groups of individuals are targeted to change behaviours in the intervention simultaneously [79]. This approach assumes that behaviour change is a group decision, and that people who regard themselves as part of a community with established norms and processes can change when the whole group changes. To apply this approach in an SSUP intervention, groups or friendship networks are identified - including networks of students who use substances within the larger network (classroom or school) – to receive interventions tailored to their needs.

The third social network approach is the induction approach which works by stimulating peer-to-peer interaction to create cascades of behavioural diffusion [79]. In the context of SSUP interventions, members in an individual’s network are persuaded to adopt a new substance use behaviour with the assumption that this new behaviour will spread. It is often considered as a combination of segmentation interventions and individual “peer leader” approaches because leaders are identified in each friendship network to deliver the intervention. To apply this approach in an SSUP intervention, peer leaders are identified from their friendship networks using nominations, and trained on how to facilitate group discussion/interaction and incorporate anti-substance use norms in their friendship networks [80]. Regardless of the social network approach chosen in an SSUP intervention, the active involvement of students in decision making is crucial to their success [29].

#### School environment.

It is important for substance use prevention interventions to include a component that addresses the broader social environment in which school-going young people are embedded. This is partly because the environment can offer positive role models and reinforcements that will maximize long-term behaviour change by making desirable substance use prevention behaviours habitual [40,81]. Indeed, Flay [81] posited that for school-based substance use prevention interventions to be effective, a supportive environment that reinforces the newly changed attitudes, normative beliefs, and social skills towards substance use prevention is needed.

Another key dimension in the school environment component is its stability or consistency in fostering and enabling recurring cues that facilitate long-term substance use prevention habits. The interest holders recommended two approaches to enhancing or creating a school environment that supports substance use prevention. The first includes the availability of leaflets and posters on substance use within schools that serve as recurring substance use prevention cues in the school environment. Students can design these posters, with guidance from teachers, researchers, and other interest holders.

*“We can also get things like leaflets, posters, and videos on substance abuse in schools... And we have to ensure that students don’t bring drugs to school because now, some of them bring it to school and share with their friends”*. (Participant L, young person)

Second, they recommended the creation of a substance-free school environment. It is well-established in the literature that access and proximity to substances in the school environment affects young people’s substance use behaviour [82–84]. Interviews with young people in step 3 revealed that some students had access to substances within the school environment. By creating a substance-free environment in schools, access to these substances will be reduced, thereby reducing the risk of substance use.

#### Health workers.

Healthcare providers can play a major role in substance use prevention interventions because they are trusted and have a unique opportunity to influence health behaviours of their patients, including those related to substance use [85]. Therefore, the interest holders recommended the need to get key health professionals such as mental health professionals involved in SSUP interventions. Three key opportunities were identified.

*“Health workers are also part; we sometimes bring them to the schools to educate the students on harmful effects of substance use. so, I think the health workers are also the next thing to think of…*(Participant E, Policy maker). *They (health workers) are also needed to help with referrals and detox for students who are addicted to drugs”.* (Participant B, NGO).

First, they recommended that mental health workers be brought into the school setting on a periodic basis to speak to young people, with a specific emphasis on motivational interviewing [86]. Motivational interviewing can help students examine their motivations for substance use, recognize the risks, and encourage healthy behaviour change. Second, health workers can use these opportunities to screen for risk factors at the early stages by means of brief surveys, in-person interviews, drop-in stations or anonymous contact with young people. Routine screening for substance use risk factors enable timely intervention before substance use escalates [87]. Lastly, interest holders recommended the creation of opportunities for students who are addicted to substances to access the need services from an appropriate healthcare team through referrals. Findings from the social network study (step 3) indicated that some school-going young people already use substances and may benefit from the referral to healthcare services. Key to the healthcare component of SSUP interventions is a referral network with community-based organizations and healthcare facilities that provide mental health and substance use services. This will enable students who need intensive support to access the needed services safely [87].

### Delivery agents

Another priority concern identified by interest holders was the need to carefully consider appropriate intervention delivery agents for different intervention components. One of the priority groups identified was teachers. Not only are teachers trusted and respected by students and parents alike; their sustained contact with students within and beyond the classroom make them key delivery agents across multiple SSUP components inclusive of education, parents and teachers, peer ambassadors, and the school environment.

*“For the school setting, the teachers will be key in the delivery, because we train a lot of young people every year…”.* (Participant C, teacher)

Peer ambassadors or peer leaders were also noted as important delivery agents given the view that young people know themselves better; and are hence well-equipped to influence each other. Their involvement is especially relevant for education, peer ambassadors, and school environment components. It is important that peer leaders are properly selected and trained for intervention delivery based on their demonstrated potential for responsible action with minimal teacher direction [88].

*“…so, if we get student ambassadors in schools and train them, they will go to their colleagues and speak to them to address drug use problems”.* (Participant G, young person).

The last group recommended for intervention delivery was counsellors and/or mental health professionals. Interest holders from education, social welfare, and community sectors reflected on their positive experience collaborating with health workers to deliver health education to young people in schools. Their involvement would be important to counselling, health workers, and education components.

*“we sometimes bring them (health workers) to the schools to educate the students on harmful effects of substance use. so, I think the health workers are also the next thing to think of…*(Participant E, Policy maker).

Given the tendency for teacher-facilitator attrition when they are the only deliverers of the intervention and not compensated, a multi-delivery group is recommended in which the core delivery agents (teachers, peer ambassadors and health workers) combine efforts to deliver the intervention. Beyond these core delivery agents, other agents can be appropriately involved in supporting SSUP interventions. Some of these agents include parents and families, health education specialists, community organizations and non-profits, governmental agencies responsible for educational activities, social welfare and community development workers (social workers in some settings), law enforcement agencies, and faith-based and religious organizations.

### Interest holder engagement

Interest holders include individuals targeted by the intervention, those involved in the development and delivery, and those who have an interest in the issue. Meaningful engagement with interest holders at each phase of the intervention will enhance the possibility of a successful intervention [89]. Also, support from interest holders is regarded as essential for the longer-term sustainability of prevention programs [78]. Throughout the deliberative dialogue, recognition of interest holder engagement as an important aspect of designing and implementing complex interventions was apparent.

Community leaders, including chiefs and other traditional leaders were identified as important interest holder group as they play a key role in the local government and health and development activities in Ghana. With respect to religious leaders, the NGO partner reflected on their experience engaging them in efforts to help people who are addicted to drugs and recommended their engagement in SSUP interventions. Other participants confirmed the potential role of religious leaders in promoting and supporting interventions and policies that improve health behaviours and outcomes, including substance use behaviour.

*“The fact of the matter is, I’m a religious leader and I’ve been talking to another religious leader about it (substance use problem) and we talk about how best we can help them (people who use substances) to come out of it... So, it is key to consider the religious leaders in prevention activities”*. (Participant B, NGO).

Participants further recommended the inclusion of policy makers and planners from the education sector, including school principals, municipal/district, regional, and national directors of education, school health education project coordinators, given their influence on the implementation and scale up of SSUP interventions. Relevant community organizations and NGO partners were also noted, given their historic role in promoting health in Ghana and other African settings. Lastly, there is the need to engage government agencies that oversee education, welfare, and community development.

*The policy makers and planners in the education sector is also important. When you wrote the letter to the Director, if she refused to allow you to carry out the study, you wouldn’t have been able to do this. so, I think they’re also interest holders that need to be brought on board.* (Participant C, teacher).

In engaging interest holders, it is important for researchers and program developers to be mindful of potential conflicts of interest and be transparent to prevent or mitigate any negative impacts. Interest holder engagement should not only elicit interest holder priorities, but also consider why they are priorities [90]. It is also important to decide on which interest holders to engage and when to engage them. The engagement process can be guided by the interest holder power-interest grid [91], with most energies focused on interest holders with high power and high interest.

The engagement of core interest holders such as young people, teachers, and policy makers and planners from the education sector is particularly useful at early stages of the SSUP intervention planning. However, challenges may be encountered in bringing young people and adults together given power imbalances that privilege the latter. To counter these imbalances, strategies are needed to elevate the voice of young people and ensure that their participation is meaningful and safe. Empowerment and coaching activities for youth are examples of how equitable participation in SSUP interventions can be enabled.

### Application of behavioural theories, models, and frameworks

Behaviour change, including substance use is a complex phenomenon and requires a theoretical understanding of the behaviour, as well as what works and under what circumstances. As a result, the incorporation of evidence-based theories, models and frameworks (TMF) are recommended in the design, implementation, and evaluation of SSUP interventions. Evidence suggests that behavioural interventions are more effective if grounded and guided by appropriate behavioural TMFs [92]. Researchers have indicated that TMFs can inform intervention development by helping identify and target the precursor of the behaviour and determinants of change [93], as well as understand and support the interventions’ mechanism of action [94]. In doing so, it is possible to determine specific factors that account for ineffective interventions, and to refine them accordingly [94].

The appropriate selection and application of a TMF is therefore a crucial step in designing a rigorous SSUP intervention, and its necessary components. Also, the resulting intervention should target relevant constructs of the chosen TMF. For instance, if an SSUP intervention aims to change substance use norms among students, the social norms theory can be applied to target a normative belief mediator. Based on this analysis, the selection of appropriate SSUP intervention components such as education, or peer ambassadors and their associated intervention approaches can be justified. Also, based on the targeted mediator of the theory, topics on perceived use, perceived acceptance, and peer pressure can be selected in the education component to educate students. In another example, if the SSUP intervention targets substance use as a problematic behaviour, the theory of problem behaviour can be applied to design and implement the SSUP intervention. Within this approach, concepts of the intervention can be used to target skills that mediate problematic substance use behaviour change. The education component of SSUP intervention might therefore target key skill building topics such as refusal skills, assertiveness, communication skills, problem solving, decision making.

An overview of a range of TMFs and approaches to their application in school-based substance use prevention interventions has been presented by Cadri and colleagues [23]. This is complemented by Michie et al. [93] who offer a scientific approach for selecting and linking a behaviour change theory to inform the design and evaluation of behaviour change interventions. Michie’s theory coding scheme [95] is particularly useful in assessing the extent to which a TMF has been appropriately applied to the design, implementation, and evaluation of an SSUP intervention.

## Discussion

This research fills an important gap in the literature by providing a culturally appropriate, context specific framework that can guide the design and adaptation of school-based substance use prevention interventions in Ghana, with potential applications in other African settings. The underlying causes of substance use behaviour goes beyond simple risk and protective factors to include a complex interplay of neurological, environmental, and social determinants that influence the development of the adolescent brain and its capacity to manage conflicting emotions and progress towards self-regulation [66]. This framework is not intended to design, adapt and/or implement an intervention that will alter these mechanisms; rather, it serves as a guide to designing strategies that enhance information, skills, networks and norms, that help prevent harmful substance use among young people.

This framework, consistent with frameworks such as the UNODC’s *International Standards on Drug Use Prevention* and WHO’s *School Health Programmes*, emphasizes evidence-based content, life skills training, and multi-sectoral collaboration. However, the Ghana-specific framework emphasizes three key respects. First, it encourages integration with existing education sector policies, such as the Ghana Education Service’s School Health Education Programme, ensuring alignment with national priorities and available infrastructure. Second, the framework embeds community, guardians, and friendship networks as central components, reflecting local friendship network and sociocultural norms where friends, family and community elders play influential roles in young people’s behaviour. Third, it addresses contextual resource constraints by prioritizing low-cost, scalable delivery mechanisms (e.g., peer and teacher-led sessions) and integrating substance use prevention into broader health promotion activities. These priorities, informed directly by stakeholder deliberations, highlight how the Ghanaian context shapes both the components and the delivery of interventions, potentially offering a model that is both culturally appropriate and feasible for low-resource settings.

This framework encourages the systematic consideration of key principles, components, TMFs, and delivery agents when conceptualizing SSUP interventions during the early stages. Beyond ensuring that the intervention considers all necessary components, it also motivates their appropriate alignment with SSUP guiding principles, the selected TMF, and with the interest holders and delivery agents involved. Also, it serves to encourage more structured investigation of the relative effectiveness of certain strategies in addressing risk factors and behavioural determinants of substance use.

While we identified a variety of components and delivery agents in the SSUP framework, some components may be prioritized depending on context specific realities and resources. Nevertheless, a basic premise of the SSUP framework is to advocate for a social network approach in SSUP interventions. In recognition of this, we regard education, parents and teachers, and peer ambassador components as particularly vital. On the educational component, the topics we highlighted are not exhaustive. Topics and content should not be chosen just because they are commonly covered in prevention interventions; but based on context-specific realities. A needs assessment may be helpful to understand these realities, including what substances are available and levels of use. Most importantly, we recommend a co-design approach to decide what content is needed, and which components are feasible and appropriate to specific contexts and settings.

The co-design approach will require meaningful engagement of interest holders at each stage of the intervention process. It is particularly important that researchers, program developers, implementers, and planners ensure that young people are safely engaged in SSUP design and implementation. In bringing young people and older interest holders together, active efforts to minimize power imbalances between young people and adult interest holders are key [96]. Also, in engaging young people, researchers, program developers, implementers, and planners need to be cognisant of critiques about the involvement of young people in research and decision making and adopt approaches to address these critiques in their SSUP intervention.

Despite the potential of the framework in reducing substance use burden among young people in Ghana, potential challenges may be encountered in its application. As such, in developing the framework, we explicitly considered real-world challenges that may serve as barriers to its implementation. Insights from the interest holder consultation and further document review highlighted key practical challenges, including resources constraints**,** inconsistent teacher training on life skills and health topics, and interest holder buy-in from different levels**,** including the district and community levels.

In resource constraint settings such as Ghana, limited budget for the implementation of an SSUP intervention can be a major challenge to its implementation [97]. In most cases, there is an over-reliance on donor-driven short-term funding for these interventions; but these are not sustainable in the long-term. Integrating an SSUP intervention within existing school health programmes such as the SHEP in Ghana, as well as advocating for a co-financing with the health, education, and youth development sectors in Ghana can be a strategy to minimize the potential budgetary constraints.

Another potential challenge relates to limited knowledge or training for teachers on substance use in schools in Ghana, which can limit their confidence in addressing this sensitive topic. Teachers will play vital roles in potential SSUP interventions in Ghana. Specifically, they are identified in three priority areas: components, delivery agents, and interest holder engagement. To address this, the framework emphasizes comprehensive training on substance use prevention, as well as ongoing support that fits within the professional development framework of teachers as a strategy to mitigate this challenge [98].

A lack of government and other interest holders’ buy-in and prioritization of SSUP interventions are also major barriers to the implementation of SSUP interventions. To address this challenge, the framework encourages engagement of relevant policy makers and other interest holders early in the planning stages of the SSUP intervention [99]. The alignment of SSUP interventions with existing national education, health, and youth development policies in Ghana may further serve to enhance policy makers’ buy-in [99].

Lastly, substance use is broadly perceived as a moral failure and has associated stigma in Ghana [45]. This can lead to parents or teachers denying the existence of the problem and/or to the resistance of certain schools in adopting substance-use prevention interventions. To address this potential challenge, it is recommended to use communication approaches that highlight substance use as a social and public health issues. Moreover, involving parents, teachers, and other local interest holders in the early stages of planning (e.g., through orientation sessions, parents and teachers association meetings, and community forums) can enhance local ownership.

### Limitations

Our framework has several limitations. First, although it is evidence-informed and constructed using a systematic process, it has not been empirically tested. While we hope that the framework will effectively guide the design and adaptation of school-based substance use prevention interventions, we have not examined the feasibility, usability or effectiveness of an intervention designed and implemented using the SSUP framework, nor assessed related implementation challenges. Also, we have not developed implementation tools to guide the practical application of the framework. As a next step, we intend to pilot the framework and develop a suite of specific implementation tools and guidelines to accompany the framework.

Another limitation relates to the contextual specificity of the deliberative dialogue. The larger social context within which the dialogue took place, and the values, experiences and perspectives of participating interest holders, no doubt influenced what was discussed and recommended. Further, there was no representation of high-risk youth (e.g., out-of-school or street-connected) and there is a potential social desirability bias in youth consultations. While evidence-based, our findings do not represent absolute generalizable knowledge. Finally, the process of developing the framework occurred solely in Ghana. It’s potential relevance to similar African settings needs to be examined.

## Conclusions

In collaboration with interest holders, we have co-developed a framework that can guide the design and adaptation of school-based substance use prevention interventions in Ghana, with potential applications in similar African settings. Beyond addressing an important gap in local implementation guidance, the framework offers a practical, flexible tool that relevant actors in Ghana can use to plan, adapt, and evaluate substance use prevention efforts in diverse school settings.

The SSUP framework can promote systematic discussion among substance use prevention scientists and inform real-world intervention development by helping decision-makers identify context-specific needs, select appropriate strategies, and engage key actors- including teachers, students, and policy makers- in co-designing culturally relevant solutions. Its modular nature allows for integration into existing school health programs and alignment with national education policies.

To realize its practical applicability, future research will focus on piloting the framework across varied school environments to assess its feasibility, usability, and impact on youth substance use outcomes. Evaluating its adaptability across age groups, genders, and levels of substance use will also be critical. By enabling structured, context-sensitive approaches to intervention design, the SSUP framework lays a foundation for more sustainable and effective prevention responses across the African region.

## Supporting information

S1 File Deliberative dialogue discussion guide. (DOCX)

S2 File Inclusivity in Global health Research checklist. (DOCX)
