# Supplementary material for: Co-developing a framework to guide school-based substance use prevention (SSUP) interventions in Ghana
Source: PLOS Glob Public Health. 2026 Feb 18;6(2):e0004345. doi: 10.1371/journal.pgph.0004345 (PMC12915948; doi:10.1371/journal.pgph.0004345)
Supplement: S1 File — (DOCX) [file pgph.0004345.s001.docx]

Supplementary file 1

| **Agenda item** | Activity | Time allocation |
| --- | --- | --- |
| **Team arrives early** | - Arrive at 9:00 - Set up breakout groups - Assign people to breakouts - Greet people as they come and make them feel welcome |  |
| **Consent** | - Ask participants if they have questions on the information sheet and get them to sign the consent forms |  |
| **Welcome and rules of the game** | - Introduce team members - Go over agenda - session etiquette: respect for everyone, don’t interrupt when someone is talking - You will need paper and pens to jot down thoughts before sharing. - Trouble shoot with Nicole or Maddie - There will be times you may feel pushed - You may feel pushed, we want to make best use of your time - Feel free to express yourself if your language of choice | 10:00 to 10:05 |
| **Introduce yourselves** | - This is a chance for us to get to know each other. Take 1 minute each to tell the others:   - your name   - your job/school | 10 mins |
| **Intro to deliberation process** | - Briefly introduce substance use prevention intervention. - Briefly introduce the goal of the project - Briefly highlight findings from the survey and IDIs - Introduce goal of Deliberative dialogue | 10 mins |
| **Let it live** | What would an ideal school-based substance use prevention intervention look like in your setting?  Based on your experience, how important will an intervention be?   - [1-2]  jot down your best idea for how this could work - [5-7,] Facilitated round table, approx. 1min per person, building on contribution or the other. Until no new ideas. | 20 minutes |
| **Let it live summing up and reflection** | - Synthesize what you heard and reflect what you see as people create a space for hope. - Ask “how do you feel”? |  |
| **Intervention** | - Brief presentation on components of intervention - Split into 2 groups - Take 1 minute to reflect individually on each questions and share ideas in a roundtable | 30 mins |
| **Questions** | - Of the key components of effective interventions, which ones would work in our locality? List and rank - For each component listed, how can we make it work in our intervention? - Which group can deliver intervention? List and rank - what are your thoughts on students actively involved in intervention delivery? - What things do we need to make such an approach work well? - Which stakeholders will be key to intervention success? - For each stakeholder listed, what role can they play to support young people in delaying the onset and reducing the rate of substance use? - How can we counter the influence of gender stereotype on substance use in the intervention - How can we reduce friendship influence on substance use in the intervention? |  |
| **Intervention sum-up in main group** | - Please provide a summary of the discussion in your group? - Does any group member want to add anything? |  |
| **Minimum specifications** | - Identify the most appropriate elements for a school-based substance use prevention intervention that actively involves students to work well in our setting - MUST HAVES & ESSENTIAL BUT NOT NECESSARY   **Sequence of Steps and Time Allocation**   - - Generate list of all must-have/do (or must not)   - Consolidate ideas from the group to make as complete a Max Specs list as possible in a short time (6 min). Consider facilitated round table: each saying one, until no new ideas (“pass”) or time runs out.   - Clarify what is not clear or points that can be consolidated   - Test each Max Spec by asking “*is this spec essential for the purpose of …”*  If the spec can be violated and the purpose achieved, then drop spec doesn’t go on Min Spec. space.   - Summarize the purpose and min specs. | 30 mins |
| **Next steps** | - What will be done next |  |
| **Thanks** |  |  |
